# A novel model of demyelination and remyelination in a GFP-transgenic zebrafish

**DOI:** 10.1242/bio.201410736

**Published:** 2014-12-19

**Authors:** Yangwu Fang, Xudan Lei, Xiang Li, Yanan Chen, Fei Xu, Xizeng Feng, Shihui Wei, Yuhao Li

**Affiliations:** 1 Key Laboratory of Tumor Microenviroment and Neurovascular Regulation, Nankai University School of Medicine, Tianjin 300071, China; 2 State Key Laboratory of Medicinal Chemical Biology, College of Life Science, Nankai University, Tianjin 300071, China; 3 Department of Ophthalmology, Chinese PLA General Hospital, Beijing 100853, China

**Keywords:** Demyelination, Myelin basic protein, Oligodendrocyte, Remyelination, Transgenic zebrafish

## Abstract

Demyelinating diseases consist of a variety of autoimmune conditions in which the myelin sheath is damaged due to genetic and/or environmental factors. During clinical treatment, some patients undergo partial remyelination, especially during the early disease stages. However, the mechanisms that regulate demyelination remain unclear. The myelin structure, myelin formation and myelin-related gene expression are highly conserved between mammals and zebrafish. Therefore, the zebrafish is an ideal model organism to study myelination. In this study, we generated a transgenic zebrafish Tg(*mbp:nfsB-egfp*) expressing a fusion protein composed of enhanced green fluorescent protein (EGFP) and NTR from the myelin basic protein (*mbp*) promoter. Tg(*mbp:nfsB-egfp*) expressed NTR-EGFP reproducibly and hereditarily in oligodendrocytes along the spinal cord. Treatment of zebrafish larvae Tg(*mbp:nfsB-egfp*) with metronidazole (Mtz) resulted in the selective ablation of oligodendrocytes and led to demyelination, accompanied by behavioral changes, including decreased total movement distance, velocity, total movement time and fast movement time. After withdrawal of Mtz for a seven day recovery period, the expression of EGFP and MBP protein was observed again which indicates remyelination. Additionally, locomotor capacity was restored. Collectively, Tg(*mbp:nfsB-egfp*), a heritable and stable transgenic line, provides a novel, powerful tool to study the mechanisms of demyelination and remyelination.

## INTRODUCTION

The myelin sheath is the membrane structure protecting, supporting and nourishing axons. As the multi-layered insulating structure around the axon, the myelin sheath mediates the rapid conduction of nerve impulses (Almeida et al., 2011; Czopka et al., 2013). Demyelinating diseases are a variety of diseases in which the neuronal myelin sheath is damaged due to genetic, environmental factors or unknown factors. The mechanisms of demyelination remain unclear. This damage impairs the conduction of signals in the affected nerves. Then, the reduction in conduction causes deficiencies in sensation, movement, cognition, or other functions, depending on which nerves are involved (Compston and Coles, 2002; Scherer and Wrabetz, 2008). The zebrafish is an ideal model to study injury and regeneration of the myelin sheath. The contributive features of zebrafish include external fertilization, large numbers of spawn, transparent eggs and embryos, rapid development, and the opportunity for genetic analysis. Moreover, the myelin structure, myelin synthesis and gene expression are highly conserved between zebrafish and mammals (Chung et al., 2013; Gerlai, 2011; Jung et al., 2010).

The Tol2 transposon is a unique autonomous transposable element in vertebrates that carries 11 kb of exogenous insert to complete the transposition without reducing the transpositional activity (Kawakami, 2005; Kawakami and Shima, 1999; Parinov et al., 2004). The nitroreductase (NTR)/metronidazole (Mtz) system is a hybrid chemical-genetic cell ablation technology widely used in zebrafish studies. This system relies on the NTR-mediated conversion of a non-toxic pro-drug (a nitroimidazole substrate, such as Mtz) into a cytotoxic agent, which induces the death of NTR positive cells, thus allowing controllable and reversible ablation of target cells (Curado et al., 2008). Moreover, Mtz can be efficiently washed out, the affected tissue can quickly recover. Therefore, the NTR/Mtz system is effective for cell ablation in a temporal and spatial manner and is applicable to any target cell population without a “bystander effect”. This technique offers a novel way to analyze tissue interactions, cell interactions, and mechanisms underlying degeneration and regeneration.

In this study, we generated a transgenic zebrafish line that expressed a fusion protein composed of enhanced green fluorescent protein (EGFP) and NTR from the *myelin basic protein* (*mbp*) promoter. Tg(*mbp:nfsB-egfp*) expressed NTR-EGFP in the oligodendrocyte lineage cells. Treatment of zebrafish larvae Tg(*mbp:nfsB-egfp*) with Mtz resulted in the selective ablation of cells expressing the NTR-EGFP fusion protein. The cell death of oligodendrocytes led to demyelination, accompanied by behavioral changes, including decreased total movement distance, average speed, total movement time and fast movement time. Strikingly, after withdrawal of Mtz, the expression of EGFP was observed again, demonstrating that remyelination occurred. Additionally, exercise capacity was partially restored. To our knowledge, this study is the first insight into the behavioral tests related to demyelination and remyelination in a zebrafish model.

## RESULTS

### *mbp* promoter-driven oligodendrocytes express EGFP

**Fig. 1.**
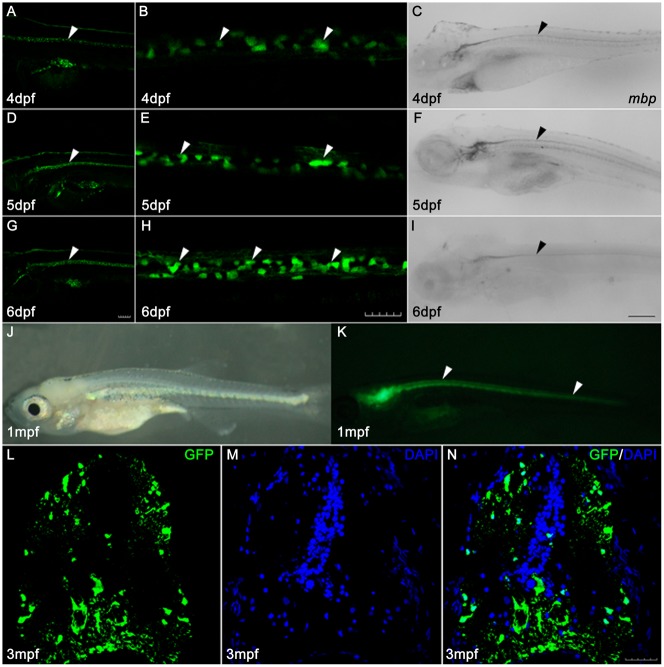
The EGFP of Tg(*mbp:nfsB-egfp*) is specifically expressed in oligodendrocytes along the spinal cord. Panels A,D,G are images of EGFP expression in larvae at 4, 5 and 6 dpf, respectively. Panels B,E,H are high magnification images of EGFP expression in spinal cords in panels A,D,G, respectively. Note that the distribution of EGFP signals continued linearly along the spinal cord (white arrowheads). Panels C,F,I show the expression of *mbp* mRNA by whole mount *in* situ hybridization in larvae at 4, 5 and 6 dpf, respectively. Note that the *mbp*-expressing cells are localized along the spinal cord (black arrowheads). Panels J,K are images of bright field (J) and fluorescence (K) of Tg(*mbp:nfsB-egfp*) at 1 mpf. EGFP continues to strongly express along the spinal cord (white arrowheads in K). Panels L–N illustrate GFP staining on sections taken from the transverse spinal cord of the 3-month-old (3 mpf) adult Tg(*mbp:nfsB-egfp*). Note that the GFP-positive cells are mainly located in dorsolateral and ventral spinal cord. Dorsal is up and rostral is left in Panels A–K. Dorsal is up in Panels L–N. Scale bar: A,D,G, 100 µm; B,E,H,L–N, 30 µm; C,F,I, 200 µm.

Myelin basic protein, encoded by the *mbp* gene, is the main protein component of the myelin sheath. The *mbp* gene is specifically expressed in differentiated oligodendrocyte lineage cells, including oligodendrocytes in the central nervous system and Schwann cells in the peripheral nervous system. Therefore, *mbp* was generally used to track and observe myelin as a marker gene (Chung et al., 2013; Jung et al., 2010; Kazakova et al., 2006; Li et al., 2007; Lyons et al., 2009). In this study, we generated transgenic zebrafish Tg(*mbp:nfsB-egfp*)^nk002^ which expressed EGFP at 4 dpf. The EGFP signals first appeared in the ventral hindbrain, nervus lateralis and spinal cord. The distribution of EGFP signals continued linearly along the spinal cord and nervus lateralis in the trunk (Fig. 1A,B, white arrowheads). EGFP expression in the spinal cord was enhanced at 5 dpf (Fig. 1D,E, white arrowheads) and 6 dpf (Fig. 1G,H, white arrowheads). *In situ* hybridization revealed that the *mbp*-expressing cells of Tg(*mbp:nfsB-egfp*)^nk002^ at 4–6 dpf were similarly localized compared to the EGFP signals (Fig. 1C,F,I, black arrowheads), and EGFP continued to strongly express along the spinal cord of Tg(*mbp:nfsB-egfp*)^nk002^ at 1 month post fertilization (mpf, Fig. 1J, bright field; K, fluorescence). To verify whether Tg(*mbp:nfsB-egfp*)^nk002^ was still active in adults, we examined the expression of GFP by immunohistochemistry on sections of the transverse spinal cord at 3 mpf. Most of the GFP-expressing cells were detected in the dorsolateral and ventral spinal cord (Fig. 1L–N), which matched with the location of highly myelinated axon bundles at this stage (Jung et al., 2010). Taken together, these results suggest that the EGFP of Tg(*mbp:nfsB-egfp*)^nk002^ was specifically and continuously expressed in oligodendrocyte lineage cells from larval stage to adulthood.

### Mtz targeting depletes oligodendrocytes of Tg(*mbp:nfsB-egfp*)^nk002^

**Fig. 2.**
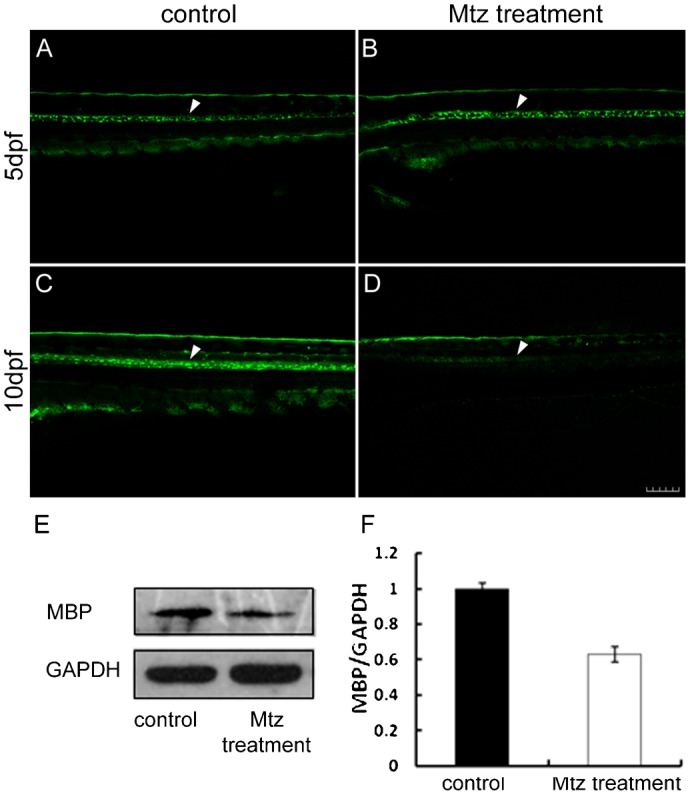
Mtz treatment specifically depletes oligodendrocytes of Tg(*mbp:nfsB-egfp*). Panels A,B demonstrate that EGFP is expressed in larvae from the 5 dpf control group and Mtz treatment group before Mtz treatment (white arrowheads), respectively. In control larva, EGFP was strongly expressed (C, white arrowhead) at 10 dpf. Panel D shows the EGFP expression in larva from the Mtz treatment group at 10 dpf after an exposure to 5 mM Mtz for 5 days. Note that the fluorescence signals were significantly reduced along the spinal cord (white arrowhead). Panel E is the result of a western blot with the MBP antibody at 5 dpf and 10 dpf. Note that the MBP protein expression was significantly suppressed in the Mtz treatment larvae at 10 dpf (F). Dorsal is up and rostral is left in Panels A–D. Scale bar: 100 µm.

Before Mtz treatment, EGFP expression was observed in Tg(*mbp:nfsB-egfp*)^nk002^ in the 5 dpf control group and the Mtz treatment group (Fig. 2A,B, white arrowheads). After exposing 5 dpf Tg(*mbp:nfsB-egfp*)^nk002^ larvae to 5 mM Mtz for 5 days, the treatment group had reduced fluorescence signal in the spinal cord (Fig. 2D) compared with steady EGFP expression in the control group (Fig. 2C). A western blot analysis demonstrated that the MBP protein expression was detected in larvae from both the control group and the Mtz treatment group at 5 dpf (Fig. 2E). However, following 5 days of Mtz treatment in transgenic larvae, the MBP protein expression was significantly suppressed in the Mtz treatment larvae at 10 dpf, while the MBP protein expression remained strong in controls (Fig. 2E,F). Together, the EGFP expression and western blot results indicate that exposure to 5 mM of Mtz for 5 days depleted oligodendrocytes and led to demyelination in the spinal cord of zebrafish larvae.

### Demyelination results in reduced locomotor capacity of Tg(*mbp:nfsB-egfp*)^nk002^ larvae

**Fig. 3.**
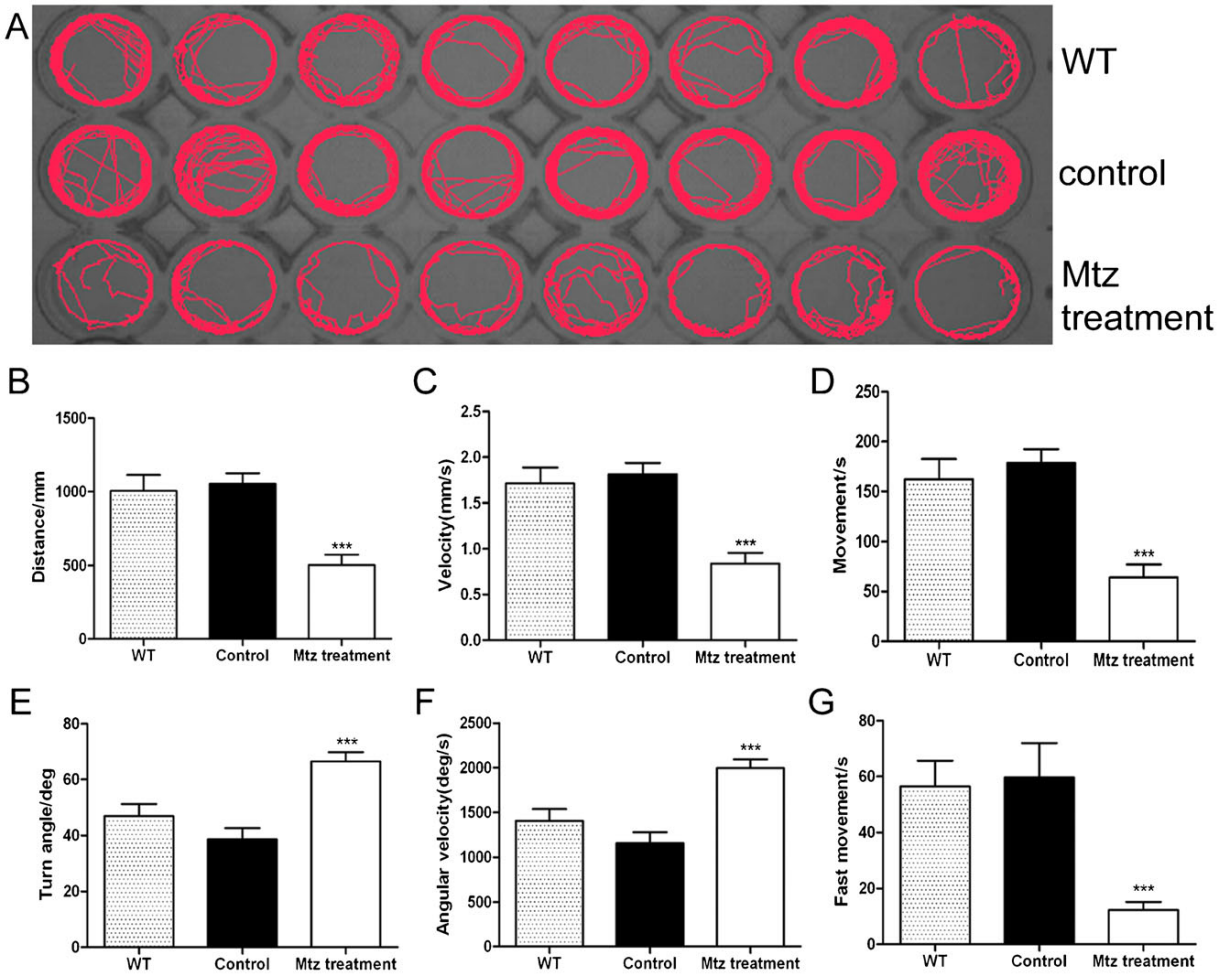
Locomotor capacity is reduced in Mtz-treated larvae of Tg(*mbp:nfsB-egfp*). Panel A is the digital tracks in larvae from wild type (WT), Tg(*mbp:nfsB-egfp*) (control) and Mtz-treated Tg(*mbp:nfsB-egfp*) (Mtz treatment) groups at 10 dpf. For each behavioral test, one larvae is placed per well, and each group has eight larvae. Panels B–G are the statistical analyses on the average of six parameters. Note that the total movement distance (B), velocity (C), total movement time (D) and fast movement time (G) in the Mtz treatment group are significantly lower than those in the WT and control groups (ANOVA, *P<0.05). However, the turn angle (E) and angular velocity (F) in Mtz-treated larvae are higher than the other two groups (ANOVA, *P<0.05).

To determine if functional changes were associated with demyelination in zebrafish, we performed behavioral tests on demyelinated larvae. There were three groups in the trial: a wild type with Mtz treatment (WT), a transgenic line without Mtz treatment (Control) and a transgenic line with Mtz treatment (Mtz treatment). The WT group served as a control for the potentially toxic effect of Mtz. After 5 days of Mtz treatment on larvae from the WT and Mtz treatment group, we performed the behavioral tests (10 dpf). The digital tracks showed that larvae from the Mtz treatment group were significantly more inactive compared with larvae from the WT and control groups (Fig. 3A). We then analyzed the digital tracks by six parameters. The total movement distance, velocity and total movement time in the Mtz treatment group were significantly lower than the WT and control groups (Fig. 3B–D, ***P = 0.001, ANOVA). More interesting, the turn angle and the angular velocity of larvae were significantly higher compared with the WT and control groups (Fig. 3E,F, ***P = 0.001, ANOVA). After Mtz treatment, the swimming time of larvae with speeds >5 mm/s was significantly shorter compared with the control group (Fig. 3G, **P = 0.0035, ANOVA). However, in all six parameters, no significant differences were found between the WT and control groups. These data also demonstrate that Mtz treatment did not have an obvious toxic effect on wild type larvae. Furthermore, these results revealed that locomotion decreased after demyelination following Mtz exposure.

### Remyelination occurs and locomotor capacity recovers in Tg(*mbp:nfsB-egfp*)^nk002^ larvae

**Fig. 4.**
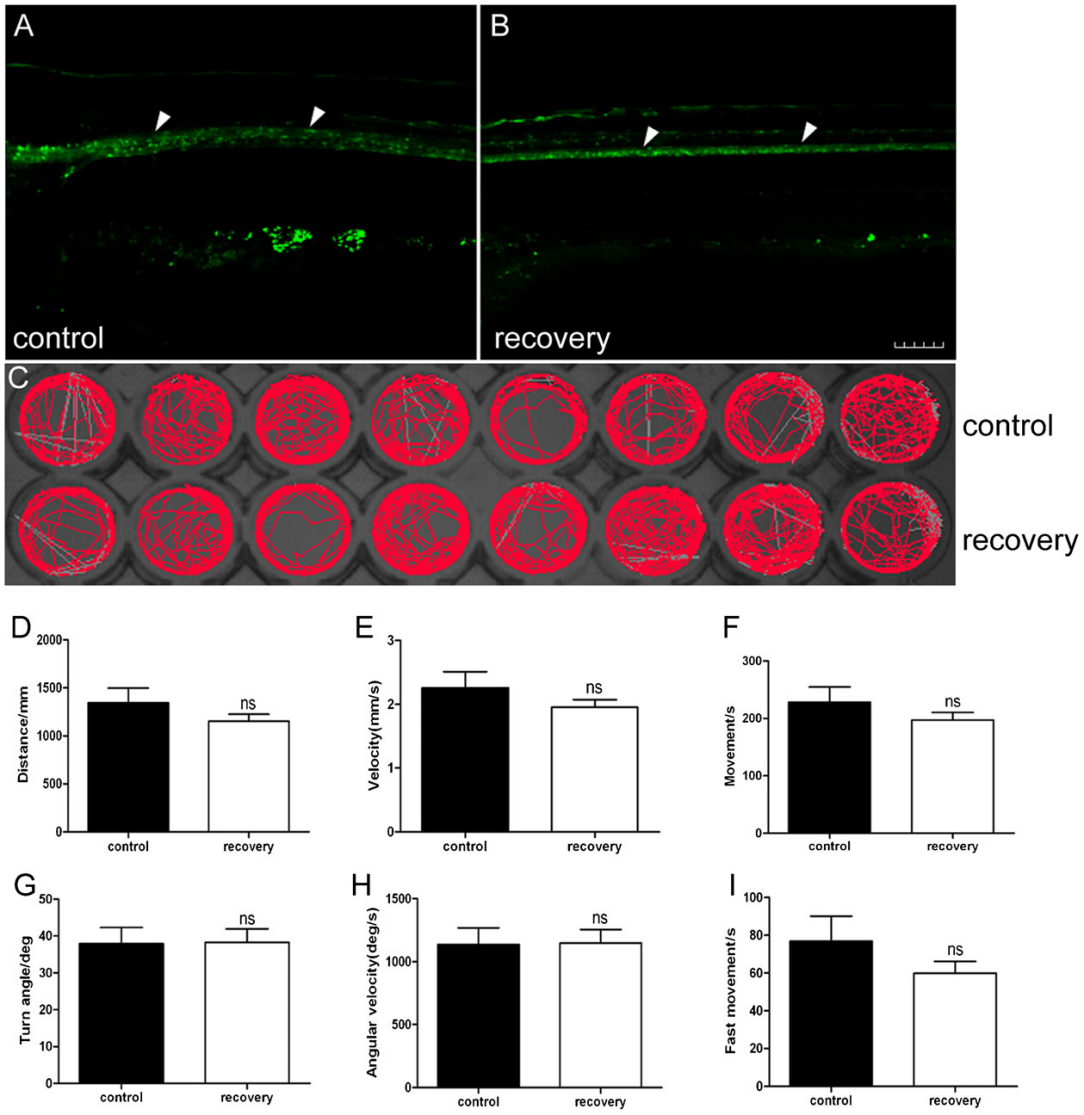
Reappearance of EGFP expression in Tg(*mbp:nfsB-egfp*) larvae results in the recovery of locomotor capacity. Panel A shows the expression of EGFP (white arrowheads) in larva from the control group at 17 dpf. Panel B shows that the expression of EGFP appeared again after a 7-day Mtz withdrawal in larva from the Mtz treatment group. Note that the positive signals are along the spinal cord (white arrowheads), which has a same expression pattern as control (A). Panel C is the digital tracks in larvae from the control group and recovery group at 17 dpf. Panels D–I show the statistical analysis on the average of total movement distance (D), velocity (E), total movement time (F), turn angle (G), angular velocity (H) and fast movement time (I) between groups. Note that there is no significant difference between the recovery group and control group (*t*-test, P>0.05). Dorsal is up and rostral is left in A,B. Scale bar: 100 µm.

To investigate the potential for regeneration following demyelination induced by Mtz, larvae were collected randomly from the 10 dpf Mtz treatment group and routinely cultured in E3 medium until 17 dpf, which was defined as the recovery group. Larvae in the control group were also collected and defined as the control group. Under the fluorescence microscope, EGFP-positive signals were observed along the spinal cord in the control (Fig. 4A) and recovery groups at 17 dpf (Fig. 4B), indicating that remyelination occurred following the seven day recovery. Behavioral tests at 17 dpf showed that the trajectory display of larvae from the Mtz recovery group was active again, which is similar to larvae from the control group (Fig. 4C). The total movement distance, the average speed, the movement time and fast movement time in larvae from the recovery group were still lower than those larvae from the recovery group, but no significant difference was found between the two groups (Fig. 4D–F,I, *t*-test, P>0.05). Additionally, the turn angle and angular velocity in larvae from the recovery group were very similar to larvae from the control group (Fig. 4G,H, *t*-test, P>0.05). These data indicate that the larvae recovered from the Mtz treatment after 7 days of regular husbandry, and regeneration of the myelin sheath occurred. Moreover, this remyelination improved the locomotor capacity.

## DISCUSSION

Myelin structure, synthesis and gene expression patterns are highly conserved between zebrafish and mammals. The myelin structure is formed by oligodendrocytes in the central nervous system and Schwann cells in the peripheral nervous system and surrounds the axons. In zebrafish, a non-tight structure is observed at 2 dpf, and the myelin sheath forms at 4 dpf. The tunica vaginalis forms a compact myelin structure at 7 dpf (Brösamle and Halpern, 2002). Most myelin-associated mammalian genes have homologies with zebrafish, including *dm20*, *mbp*, *sox10*, etc. (Emery, 2010; Farrar et al., 2011; Schweitzer et al., 2006).

Chemical methods have often been used to induce demyelination in mouse models (Merrill, 2009). For example, demyelination was induced by feeding Cuprizone for 3 weeks, and remyelination occurred after drug discontinuation for 4 weeks (Matsushima and Morell, 2001). Due to unclear drug action mechanisms, it has been difficult to simulate the human immune response and pathogenesis. Demyelination can also be induced in two weeks by injecting ethidium bromide (EB) into specific areas of the central nervous system of the mouse. However, EB is associated with collateral damage that involves other nucleated cells in the injected area and causes death of non-oligodendrocytes (Merrill, 2009). The myelination models of transgenic zebrafish includes Tg(*plp-egfp*), Tg(*olig2-egfp*), Tg(*sox10-egfp*) and Tg(*mbp-egfp*), which drive GFP by different gene promoters (Carney et al., 2006; Jung et al., 2010; Lyons et al., 2009; Yoshida and Macklin, 2005). However, injury to the myelin sheath can not be specifically induced in these transgenic models. NTR-Mtz cell ablation technology made it possible to successfully ablate the target cells (Curado et al., 2008; Kondrychyn et al., 2009; Suster et al., 2009). In this study, transgenic zebrafish Tg(*mbp:nfsB-egfp*) were generated with the EGFP gene driving the *mbp* promoter. Tg(*mbp:nfsB-egfp*) showed positive fluorescence at 4 dpf, corresponding with *mbp* mRNA (Fig. 1A–C). The signals increased in strength with the developmental period (Fig. 1D–I). The distribution of positive signals was spatio-temporally consistent with the formation of zebrafish myelin under physiological conditions (Yoshida and Macklin, 2005). The EGFP was exclusively and steadily expressed in oligodendrocytes at 1 mpf (Fig. 1K) and 3 mpf (Fig. 1L–N), showing that this line was still working until adulthood. Moreover, oligodendrocytes were specifically depleted with 5 mM of Mtz for 5 days (Fig. 2D). The GFP signals appeared again after 7 days of recovery (Fig. 4B), suggesting that demyelination in Tg(*mbp:nfsB-egfp*) was specifically drug-induced followed by secondary regeneration. Injury and regeneration have also been found in patients with multiple sclerosis, particularly during the early stage (Compston and Coles, 2002; Scherer and Wrabetz, 2008). Therefore, the transgenic zebrafish Tg(*mbp:nfsB-egfp*) model generated in this study had two advantages. First, this transgenic zebrafish constructed using NTR-Mtz technology was stable and heritable. We can easily obtain demyelinated zebrafish with the same phenotype and genetic background. Second, oligodendrocytes were specifically targeted and depleted with Mtz treatment. Because this cell ablation is reversible, this transgenic model enables the observation of demyelination and remyelination *in vivo* in the same individual. This model is a powerful tool for in-depth investigation on the mechanisms of degenerative diseases and remyelination in larvae, juveniles and adults.

Zebrafish normally prefer hyperactivity. The behavioral analysis of movement is very intuitive, simple and quick (Deshmukh et al., 2013; Lieschke and Currie, 2007; Riehl et al., 2011). Until now, the behavioral aspects of zebrafish demyelination and regeneration have not been reported. In this study, locomotion was evaluated by determining the total movement distance, velocity, total movement time, turn angle, angular velocity and fast movement time of zebrafish. Three swimming speeds, namely slow (<5 mm/s), medium (5 mm/s≤v≤20 mm/s) and fast (>20 mm/s) described the movement trajectory of juvenile zebrafish (Deshmukh et al., 2013; Winter et al., 2008). The total movement distance and velocity decreased significantly in larvae with demyelination (Fig. 3B,C), suggesting a decreased swimming ability. The time of movement and fast movement in the Mtz-treated group were significantly shorter than the control groups, indicating that most individuals preferred a motionless state following demyelination (Fig. 3D,G). To our surprise, the turn angle and angular velocity in the Mtz-treated group were higher than the control groups (Fig. 3E,F). Turn angle and angular velocity are helpful for detecting abnormalities of behavior, such as stereotypic movements and reaction to toxic substances. In zebrafish, the turn angle is the change of head direction and the angular velocity represents the amount of turning per unit time. We presumed that the turn and angular velocity increased probably because the larvae with demyelination can not control their motor direction robustly. After 7 recovery days, locomotion in the demyelinated larvae had significantly recovered and was not different from the control group (Fig. 4D–I). This finding partially simulates the pathological and functional changes associated with early multiple sclerosis clinically.

Overall, the transgenic zebrafish Tg(*mbp:nfsB-egfp*) generated in this study causes targeted demyelination and remyelination, which is accompanied by an altered locomotion pattern. Tg(*mbp:nfsB-egfp*), a heritable and stable transgenic line, provides a novel, powerful tool to investigate the mechanisms of demyelination and remyelination *in vivo* and will contribute to the comprehensive understanding of demyelinating diseases.

## MATERIALS AND METHODS

### Experimental animals

AB strain zebrafish were used in this study. The animals were maintained in aquaria at 28.5°C under a 10/14-hour dark/light cycle (Westerfield, 1993). Embryos were collected after natural spawns, housed at 28.5°C, and staged by hours post-fertilization (hpf) or days post-fertilization (dpf). Protocols for all animal procedures were approved by the Institutional Animal Care Committee at Nankai University and conformed to National Institutes of Health guidelines.

### Generation of transgenic lines

The Tol2 system was used to generate transgenic fish (Luo et al., 2012; Montgomery et al., 2010). The zebrafish *myelin basic protein* (*mbp*, GenBank AY860977) promoter was PCR-amplified with primers containing *Bam*HI and *Nco*I restriction sites. The primer sequences were: forward, 5′-CGCCGGGATCCATAATAACAATCCCAACTC-3′; reverse, 5′-CGCCGCCATGGGTATGTCCTTCTCCGCTCA-3′ (the restriction sites are underlined). PCR was conducted with the following cycling parameters: 94°C for 7 minutes; 35 cycles of 94°C for 45 seconds; 56°C for 2 minutes; 72°C for 1 minute; and a final extension of 72°C for 7 minutes. The purified PCR product was cloned into the T2KXIG-*pgrna*:nfsB-EGFP plasmid (a kind gift from Dr. Hitchcock) by a *Bam*HI/*Nco*I (Promega, Madison, WI) digest to remove the *progranulin-a* (*pgrna*) promoter and ligate the amplified *mbp* promoter. The construct contained approximately 1.9 kb of 5′ flanking sequences plus the first exon of the *mbp* gene upstream of cDNA encoding EGFP. Tol2 transposase mRNA was transcribed *in vitro* from the pCS2FA plasmid (a kind gift from Dr. Hitchcock) with the SP6 mMessage mMachine kit (Ambion, Foster City, CA).

The T2KXIG-*mbp:nfsB-egfp* construct was co-injected with Tol2 transposase mRNA into one- to four-cell stage AB wild-type embryos at a concentration of 25 ng/µl and 35 ng/µl, respectively (Montgomery et al., 2010; Thummel et al., 2005). Embryos positive for the gene construct were identified at 96 hpf by the *mbp*-driven expression of EGFP along the spinal cord and nervus lateralis. Positive F0 adults were out-crossed to the wild-type AB fish to identify founders. EGFP-expressing F1 carriers were out-crossed to generate independent transgenic lines. Two lines, Tg(*mbp:nfsB-egfp*)^nk002^ and Tg(*mbp:nfsB-egfp*)^nk003^, were characterized and found to yield approximately 50% GFP-positive progeny upon mating with wild-type AB fish. Tg(*mbp:nfsB-egfp*)^nk002^ was used for all of the data presented herein.

### *In situ* hybridization

Embryos were grown in 0.003% 1-phenyl-2-thiourea (PTU, Sigma, St. Louis, MO) to block pigmentation and mediate visualization until 6 dpf. Larvae were anesthetized with 0.1% ethyl 3-aminobenzoate methanesulfonate salt (MS-222, Sigma), euthanized immediately and fixed in 4% paraformaldehyde. *In situ* hybridization was performed on whole-mount larvae according to a standard protocol (Thisse and Thisse, 2008). Oligodendrocyte lineage cells were labeled using an mRNA probe for *mbp*. Briefly, the sense and antisense riboprobes were synthesized from linearized plasmids, and digoxigenin (DIG)-labeled probes were generated by *in vitro* transcription using the DIG RNA labeling kit (Roche Diagnostics, Indianapolis, IN). The cDNA encoding *mbp* was linearized with *NdeI*, and the riboprobes were synthesized with T7 polymerase. Whole-mount *in situ* hybridization was performed using a 2 ng/µl probe in Eppendorf tubes and hybridized overnight at 55°C. The non-hybridizing riboprobe encoding the sense strand of the respective cDNAs was used as the negative control. The next day, the embryos were washed, and digoxigenin was immunolabeled using an alkaline-phosphatase-conjugated antibody (Roche). NBT/BCIP (Roche) served as the enzymatic substrate on the third day.

### Immunohistochemistry

3-month-old adult Tg(*mbp:nfsB-egfp*)^nk002^ were anesthetized with 0.1% MS-222 (Sigma). Some of the trunk were dissected and fixed in 4% paraformaldehyde, cryoprotected in 20% sucrose in 0.1 M PBS (pH 7.4), frozen in Optimal Cutting Temperature Compound (Sakura Finetek, Torrance, CA, USA). Transverse cryosectioning at 10 µm were performed with a cryostat (Leica CM1850, Wetzlar, Germany) and mounted on glass slides. Immunohistochemistry was performed using standard procedures (Huang et al., 2012). In brief, after drying, sections were incubated with 20% normal sheep serum (NSS) in PBS containing 0.5% Triton X-100 (PBST), followed by overnight incubation at 4°C in an anti-GFP antibody (ab6556, diluted at 1∶1000, Abcam, Cambridge, MA). In the next day, sections were washed extensively with PBST, incubated in an FITC conjugated secondary antibody (diluted at 1∶500, Millipore, Billerica, MA) for 1.5 hrs at room temperature. DAPI (4′,6-diamidino-2-phenylindole, Sigma) was used as a counterstain to label the nuclei. After washing in PBST, sections were sealed with mounting media and glass coverslips.

### Western blot analysis

Western blot analysis was performed to test the expression of MBP protein. Ten larvae were lysed in buffer with protease inhibitors (Complete Mini, Roche, Mannheim, Germany), and the protein concentrations were quantified with a BCA Protein Assay Kit (Complete Mini, Roche, Mannheim, Germany). The proteins were separated in a 10% SDS-PAGE gel and were transferred to a PVDF membrane (Millipore, Billerica, MA). The membrane was then blocked in 5% nonfat dry milk in PBS for 1 hour and incubated with anti-MBP (1∶500; Anaspec, Fermont, CA) and anti-GAPDH (1∶3,000; Millipore). The blots were rinsed with PBS and incubated with peroxidase-conjugated goat anti-rabbit antibodies (1∶5,000; Sigma-Aldrich, St. Louis, MO) for 1 hour. The bound antibody was visualized using an enhanced chemiluminescence assay (Millipore).

### Metronidazole preparation and treatment

Mtz (M3761, Sigma) was dissolved in 0.2% DMSO in E3 medium with vigorous shaking and was protected from ambient light. To ablate oligodendrocyte lineage cells, Tg(*mbp:nfsB-egfp*)^nk002^ larvae at 5 dpf were placed in six-well plates containing 5 ml of 5 mM Mtz-0.2% DMSO-E3 medium for 5 days at 28.5°C in the dark. At 9 AM in each day, the larvae were allowed to have a 30-min interval in E3 medium and fed with paramecia. Then, the larvae were again exposed to Mtz solution. To eliminate the toxic effect of Mtz, 5 dpf wild type larvae were also treated with Mtz for 5 days. As controls, Tg(*mbp:nfsB-egfp*)^nk002^ larvae were incubated in 0.2% DMSO-E3 medium. After Mtz treatment, the larvae from the treatment and control groups were washed three times with E3 medium for 5 minutes each and were returned to E3 medium until 17 dpf. A minimum of ten larvae were anesthetized in 0.1% ethyl 3-aminobenzoate methanesulfonate salt (MS-222; Sigma) and identified the *mbp*-driven expression of EGFP along the spinal cord and nervus lateralis.

### Behavioral analysis

Experiments were carried out at 10 dpf and 17 dpf, respectively. At 10 dpf, the larvae were divided into three groups: wild type with Mtz treatment (WT), a transgenic line without Mtz treatment (control) and a transgenic line with Mtz treatment (Mtz treatment). At 17 dpf, there were two groups. The larvae from the Mtz treatment group were defined as the recovery group, and the larvae from control group were defined as the control group. Larvae from each group were collected, cleaned and placed in 48-well plates. Each well contained 1 ml of E3 medium and one larva, and eight larvae were in each group. Behavioral tests were performed as following: the larvae were allowed to acclimate for 15 min before locomotion monitoring (Zhao et al., 2012). Next, the larvae were allowed to freely explore the aquarium for 10 minutes. A camera positioned above the plate was used for movement tracking. All digital tracks were analyzed by Ethovision XT software (Noldus Information Technology, Wageningen, Netherlands), and a minimum movement distance of 0.2 mm filtered out system noise. Six parameters, including the total movement distance, velocity, total movement time, turn angle, angular velocity and time of fast movement were analyzed.

### Photography and image analysis

Images of the embryos positive for the gene construct were screened with a BX51 fluorescence microscope (Olympus, Japan). Images of positive larvae were photographed with an FV1000 confocal microscope (Olympus) or BX51 fluorescence microscope (Olympus). Images of the immunohistochemistry were photographed with an FV1000 confocal microscope (Olympus). Whole-mount *in situ* hybridized larvae images were photographed with a DP72 digital camera mounted on an SZX10 dissecting microscope (Olympus, Japan). The resulting images were compiled in Adobe Photoshop CS2 (Adobe, San Jose, CA, USA) and resized. They were occasionally modified for contrast and brightness using the Image-Adjustments-Contrast-Brightness setting. All of the images within an experiment were manipulated similarly.

### Statistical analysis

Statistical analysis was performed with GraphPad software (version 5.0c, GraphPad Software, La Jolla, USA). Excel was used to calculate the average values of total movement distance, velocity, total movement time, turn angle, angular velocity and fast movement time for each group. WT, control and Mtz treatment groups at 10 dpf were analyzed with a one-way ANOVA, and the control and recovery groups were analyzed with Student's *t*-test. The significance level was set at a *p* value of 0.05.
